# Exploring AI-assisted cameras to assess use of contact precautions

**DOI:** 10.1017/ice.2026.10408

**Published:** 2026-04

**Authors:** Robert J. McLuckey, Zaki Chowdhury, Katherine E. Goodman, Lisa Pineles, Michelle Newman, Gregory M. Schrank, Daniel J. Morgan, Giuliano Scarcelli, Anthony D. Harris

**Affiliations:** 1 University of Maryland School of Medicine, USA; 2 University of Maryland at College Park, USA; 3 University of Maryland Institute for Health Computing, North Bethesda, MD, USA

## Abstract

Contact precaution policies are used to prevent the spread of pathogenic organisms. We aimed to test whether AI-assisted cameras could monitor aspects of compliance with these policies. Testing in both simulated and real patient care settings yielded exceptional sensitivity and good specificity, indicating potential to monitor adherence to contact precautions.

## Background

The use of contact precautions for patients with antibiotic-resistant bacteria continues to be a controversial topic.^1–3^ Risk-based contact precautions, e.g. requiring gloves and gowns for healthcare personnel (HCP) who perform “high risk” interactions or touch the patient in an area surrounding the patient’s bed (“red zone”), have been proposed as an alternative to traditional contact precautions.^4–6^ The challenge of these policies is that they are more complex to implement and monitor compliance.

To our knowledge, no study has used video technology coupled with artificial intelligence (AI) to monitor compliance with contact precautions.

The aim of our study was to assess in a proof-of-concept trial whether AI-assisted video cameras can accurately monitor entrance into a red zone around a patient’s bed as the first step towards monitoring compliance with contact precaution policies.

## Methods

**Figure 1.**
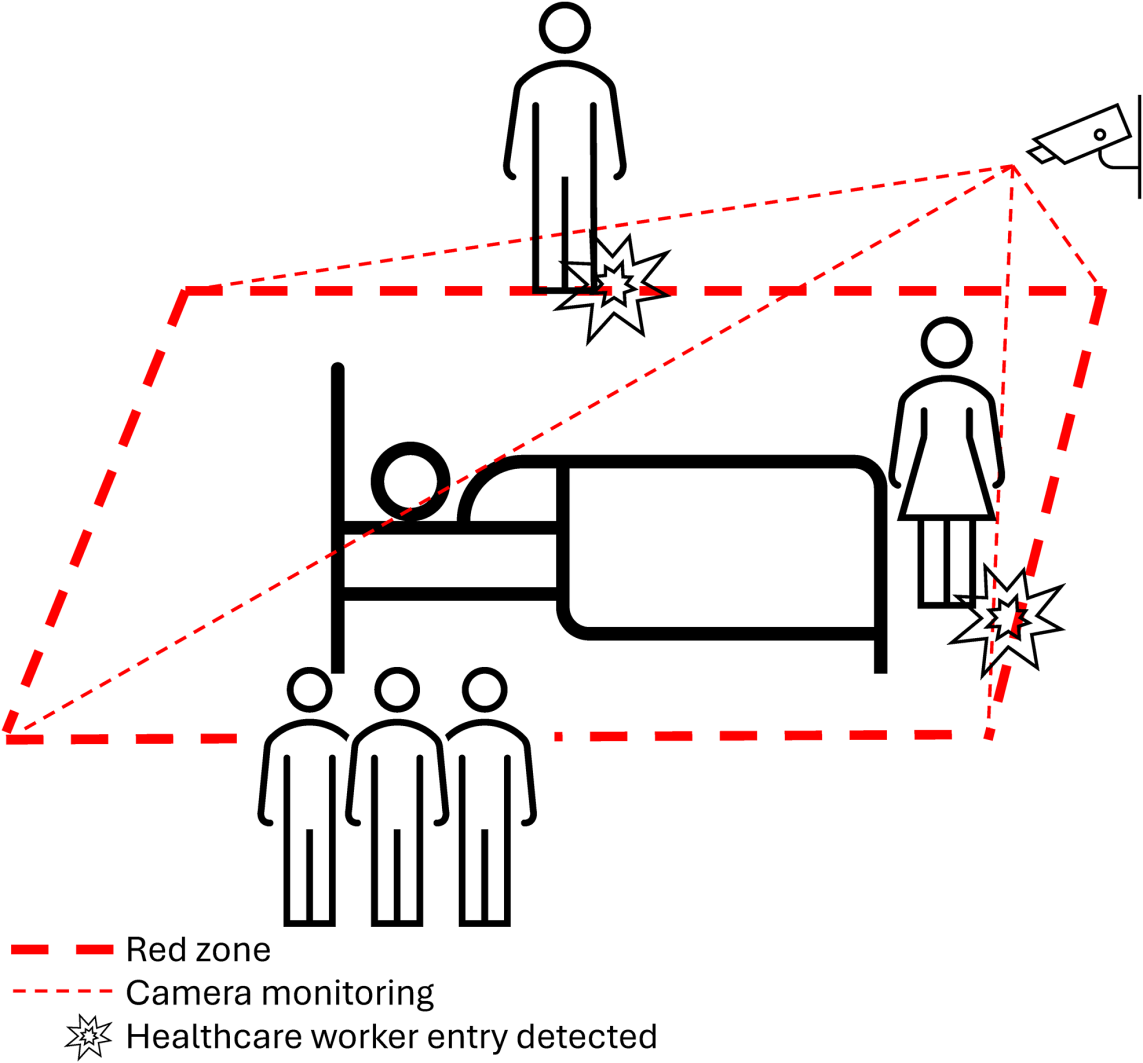
Diagram of cameras monitoring red zone.

To assess the effectiveness of AI-assisted camera technology at measuring entry into the red zone by HCP, two trials were conducted. The results obtained by the AI-assisted camera were tested against a gold standard of human observation in two phases: (1) the R.A. Cowley Shock Trauma Simulation Center (“simulation laboratory”); and (2) one patient room in the medical intensive care unit (MICU) at the University of Maryland Medical Center. The red zone was defined as a 1-foot rectangular perimeter surrounding a patient’s bed. In all settings, a camera used to monitor policy adherence was affixed to the ceiling of the room at the foot of the bed. In addition to this, the area of view in which any human faces were captured was blurred out by the camera software to maintain anonymity. The camera technology was set to monitor the red zone around the patient bed and detect HCP (Figure 1).

The imaging system utilized a standard RGB (red, green, blue) camera connected to a local computer. The area to monitor, i.e. the red zone, was defined by manually selecting four points on the video feed corresponding to the perimeter around the patient’s bed. The detection/segmentation of persons was implemented using the YOLOv8n-pose model developed by Ultralytics,^7^ a deep learning model used to identify human body landmarks including the head, wrists, hips, and ankles. To interface the detection of people and the red zone without interference, a geometric containment algorithm was used to determine whether any of an individual’s body landmarks fell within the defined boundary. If any landmarks were detected within the boundary after previously being seen outside, the individual was considered to have entered the red zone, and an entry event was recorded. Video recordings were saved locally to compare the data to the gold standard and were deleted after 48 hours.

In Phase 1, research team members interacted with a mannequin patient in a simulation room. During the encounters, both the camera and human observer monitored whether the red zone was entered. Multiple configurations/rounds of testing (single camera, double camera, different computer coding) were tested during this phase. With the two-camera setup, one camera was at the foot of the bed, and one camera was over the middle of the bed but angled to better visualize activities over the head of the bed. The scenarios were carried out by multiple individuals to account for differences in body sizes, skin tones, and movements. Based on Phase 1 results, it was determined that the most effective setup was a single camera setup positioned above the foot of the bed. This was the setup used for Phase 2.

Phase 2 was conducted in a MICU room. HCP and patients were informed of the study procedures and provided verbal consent before monitoring occurred. In Phase 2, both the camera and human observer monitored whether HCP entered the red zone. A priori, we decided to study 100 HCP room entries. We performed power calculations aiming to assess sample size needed for narrow confidence intervals of sensitivity and specificity values between 80 and 99%. We found that 95% confidence intervals would be ± 8% with a sample size of 100 observations. Following both phases of testing, the results determined by the AI-assisted camera system were compared to the results determined by the gold-standard human observer. Both sensitivity and specificity were calculated with 95% confidence intervals to determine if the system was able to accurately detect red zone entries.

This study was approved by the University of Maryland Baltimore Institutional Review Board.

## Results

No patients or HCP declined to participate. Phase 1 (simulation laboratory) results are shown in Table 1. Phase 1.1 with a single camera had poor sensitivity due to problems with false negative zone entries near the head of the bed. In Phase 1.3 with a single camera and revised AI coding, the sensitivity was .94 (95% CI, .89–0.99), and the specificity was 95% (95% CI, .91–.99).

**Table 1.** Results from phase 1 and phase 2 testing

Testing phase	Camera configuration	Sensitivity	Specificity
Phase 1 (simulation laboratory)			
Phase 1.1	Single camera	.71 (.66–.76)	.96 (.94–.98)
	Single camera (analysis excluding entries behind the head of the bed)	.85 (.82–.88)	.96 (.94–.98)
Phase 1.2	Two cameras	1.00 (.99–1.00)	.71 (.67–.75)
Phase 1.3	Single camera (additional coding)	.94 (.89–.99)	.95 (.91–.99)
Phase 2 (MICU)	Single camera	.98 (.95–1.00)	.84 (.77–.91)

Results from Phase 2 (inpatient MICU room) are also shown below in Table 1 with 95% confidence intervals in parentheses. The sensitivity was .98 (95% CI, .95–1.0) and specificity .84 (95% CI, 0.77–0.91).

## Discussion

Results showed that the AI-assisted camera technology was effective at detecting HCP entries into a zone surrounding a patient bed at a sensitivity of 98% and a specificity of 84%.

There are a number of practical issues related to the future acceptability of the use of AI-assisted cameras and wearables by HCP and patients.^8^ In our study, the acceptability was high but this was only for a specific use and short time of camera use. This acceptance by HCP was also likely driven by the fact that the use of the cameras was clearly explained as strictly for monitoring entry of the red zone close to the patient and not for monitoring other activities. Currently, AI-assisted cameras are used in day-to-day settings in the domains such as security, public safety, and traffic monitoring. They are also used in healthcare for telemedicine, EEG monitoring and “telesitter” monitoring. The future role of AI-assisted cameras in healthcare is uncertain. We believe that its potential is great but that initial usage should be focused on improving patient safety.

The next sequential step is to use AI to determine whether HCP are wearing gloves and gowns by training an image classification model on collected images and videos to automatically distinguish gloved versus ungloved hands and gowned versus ungowned attire. The coupling of this addition to the results presented in this manuscript leaves us optimistic that AI-assisted cameras offer a viable opportunity for automated monitoring of HCP glove and gown compliance. Successful recognition of gloves and gowns has already been achieved in industrial settings and in simulation laboratories, and thus we think this step is very achievable.^9,10^ In addition, AI-assisted cameras could easily be used to remind HCP in real time to don gloves and gowns or to assess masking compliance. Numerous innovative uses of AI-assisted cameras are being studied outside of the field of infection control.^11,12^

Study limitations include persistent double-counting errors where HCP were counted more than once after entering or exiting the red zone once. These arose from HCP being partially occluded, leading to incorrect AI inferences of them leaving the red zone and coming back in. This issue was resolved by implementing logic that requires a person to be consistently tracked moving from outside the zone to inside before an entry is counted, ensuring that any detection fluctuations or errors within the zone no longer result in multiple counts for the same individual. This error did not affect sensitivity or specificity but affected the accuracy of the total denominator of visits. Further limitations may also arise as the technology is tested in increasingly different settings throughout different hospitals/unit types. Further modification and testing is needed to determine the impact of these issues on future use.

In conclusion, in this pilot trial we found that an AI-assisted camera could accurately monitor entrance into red zones around patient beds in both simulation and real-world settings. The AI-assisted camera technology has potential to be used in many other areas of infection control as well as non-infectious disease quality and patient safety outcomes.

## Supporting information

McLuckey et al. supplementary material McLuckey et al. supplementary material

## Supplementary material

The supplementary material for this article can be found at https://doi.org/10.1017/ice.2026.10408.
