# Supplementary material for: Exploring AI-assisted cameras to assess use of contact precautions
Source: Infect Control Hosp Epidemiol. 2026 Feb 23;47(4):414–6. doi: 10.1017/ice.2026.10408 (PMC13216791; doi:10.1017/ice.2026.10408)
Supplement: McLuckey et al. supplementary material [file S0899823X26104085sup001.pdf]

```python
import warnings

warnings.filterwarnings("ignore", category=FutureWarning)
import cv2
import numpy as np
import os
import json
from datetime import datetime
from ultralytics import YOLO

# Loads YOLOv8 Pose Model
yolo_model = YOLO("yolov8n-pose.pt")

# File setup to save video files and boundary coordinates
output_folder = "recordings"
polygon_file = "polygon.json"
os.makedirs(output_folder, exist_ok=True)

# On screen button locations and dimensions
record_button_pos = (10, 10)
reset_button_pos = (150, 10)
button_size = (120, 40)

# Application dictionary of each tracked parameter
state = {
    'polygon_points': [],
    'mouse_position': (0, 0),
    'cross_count': 0,
    'person_trackers': {}
}

# Recording related variables
recording = False
video_writer = None

# Loads boundary if saved
if os.path.exists(polygon_file):
    try:
        with open(polygon_file, "r") as f:
            state['polygon_points'] = json.load(f)
            print("Loaded saved polygon points.")
    except Exception as e:
        print("Failed to load polygon:", e)
```

```
# Handles clicks for buttons and drawing the red zone boundary
def click_event(event, x, y, flags, param):
    global recording, video_writer
    if event == cv2.EVENT_MOUSEMOVE:
        state['mouse_position'] = (x, y)
    elif event == cv2.EVENT_LBUTTONDOWN:
        # Record button
        if record_button_pos[0] <= x <= record_button_pos[0] + button_size[0] and \
                record_button_pos[1] <= y <= record_button_pos[1] + button_size[1]:
            recording = not recording
            if recording:
                print("Recording started.")
                filename = "recording_" + datetime.now().strftime("%Y-%m-%d_%H-%M-%S") + ".avi"
                filepath = os.path.join(output_folder, filename)
                fourcc = cv2.VideoWriter_fourcc(*'XVID')
                video_writer = cv2.VideoWriter(filepath, fourcc, 10.0, (640, 480))
            else:
                print("Recording stopped.")
                if video_writer:
                    video_writer.release()
                    video_writer = None
            return
        # Reset button
        if reset_button_pos[0] <= x <= reset_button_pos[0] + button_size[0] and \
                reset_button_pos[1] <= y <= reset_button_pos[1] + button_size[1]:
            state['polygon_points'] = []
            state['cross_count'] = 0
            state['person_trackers'] = {}
            if os.path.exists(polygon_file):
                os.remove(polygon_file)
            print("Reset.")
            return
        # Drawing boundary points
        if len(state['polygon_points']) < 4:
            state['polygon_points'].append((x, y))
            print(f"Point {len(state['polygon_points'])}: {x}, {y}")
            # Saves boundary when 4 points are selected
            if len(state['polygon_points']) == 4:
                with open(polygon_file, "w") as f:
                    json.dump(state['polygon_points'], f)
                print("Polygon saved.")

# Set of helper functions for boundary detection logic
def any_point_in_polygon(points, polygon):
```

```
    # Checking if any of the landmarks are inside the zone
    if len(polygon) < 4:
        return False
    for pt in points:
        if cv2.pointPolygonTest(np.array(polygon, np.int32), (pt[0], pt[1]), False) >= 0:
            return True
    return False


def any_body_point_in_polygon(landmarks, polygon):
    if len(polygon) < 4 or len(landmarks) == 0:
        return False

    # YOLO keypoints: 0-4 are head (nose, eyes, ears), 5-16 are body
    body_landmarks = []
    for i in range(5, min(len(landmarks), 17)):
        if landmarks[i][0] > 5 and landmarks[i][1] > 5:  # Valid landmark coordinates
            body_landmarks.append(landmarks[i])
    # Returns true if any point is within the boundary
    for pt in body_landmarks:
        if cv2.pointPolygonTest(np.array(polygon, np.int32), (pt[0], pt[1]), False) >= 0:
            return True
    return False

def is_person_completely_outside_zone(landmarks, polygon):

    if len(polygon) < 4:
        return True
    if any_body_point_in_polygon(landmarks, polygon):
        return False
    return True

# Facial blur using head keypoints
def blur_head_from_landmarks(frame, landmarks):
    head_points = [(int(x), int(y)) for i, (x, y) in enumerate(landmarks) if i in [0, 1, 2, 3, 4] and x > 5
and y > 5]
    if len(head_points) >= 2:
        x_coords = [p[0] for p in head_points]
        y_coords = [p[1] for p in head_points]
        x_min, x_max = max(min(x_coords) - 10, 0), min(max(x_coords) + 10, frame.shape[1])
        y_min, y_max = max(min(y_coords) - 40, 0), min(max(y_coords) + 40, frame.shape[0])
        roi = frame[y_min:y_max, x_min:x_max]
        if roi.size > 0:
            blurred = cv2.GaussianBlur(roi, (51, 51), 30)
```

```
            frame[y_min:y_max, x_min:x_max] = blurred
    return frame

# YOLO detection function
def detect_people_and_landmarks(frame):
    results = yolo_model.predict(frame, imgsz=320, conf=0.30, iou=0.15, verbose=False)
    people = []
    for r in results:
        if r.boxes is not None:
            boxes = r.boxes.xyxy.cpu().numpy()
            keypoints = r.keypoints.xy.cpu().numpy() if r.keypoints is not None else [None] *
len(boxes)
            for box, kps in zip(boxes, keypoints):
                x1, y1, x2, y2 = map(int, box)
                body_landmarks = [(int(x), int(y)) for x, y in kps] if kps is not None else []
                people.append({'box': (x1, y1, x2, y2), 'landmarks': body_landmarks})
    return people

# Initialize camera feed
cap = cv2.VideoCapture(0)
cap.set(cv2.CAP_PROP_FPS, 30)

cv2.namedWindow('Camera View')
cv2.setMouseCallback('Camera View', click_event)


# Main loop
while cap.isOpened():
    ret, frame = cap.read()
    if not ret:
        continue
    frame = cv2.flip(frame, 1)
    frame = cv2.resize(frame, (640, 480))

    detections = detect_people_and_landmarks(frame)
    new_person_trackers = {}

    for person in detections:
        x1, y1, x2, y2 = person['box']
        landmarks = person['landmarks']

        # Draw bounding box
        cv2.rectangle(frame, (x1, y1), (x2, y2), (0, 255, 255), 2)
```

```
        # Blur head for privacy
        frame = blur_head_from_landmarks(frame, landmarks)

        # Draw skeleton
        skeleton_pairs = [
            (5, 7), (7, 9), (6, 8), (8, 10), (5, 6), (5, 11), (6, 12),
            (11, 12), (11, 13), (13, 15), (12, 14), (14, 16)
        ]
        for (start, end) in skeleton_pairs:
            if start < len(landmarks) and end < len(landmarks):
                pt1, pt2 = landmarks[start], landmarks[end]
                if (pt1[0] > 5 or pt1[1] > 5) and (pt2[0] > 5 or pt2[1] > 5):
                    cv2.line(frame, pt1, pt2, (0, 255, 0), 2)

        # Draw keypoints (excluding head points for privacy)
        for idx, (lx, ly) in enumerate(landmarks):
            if lx > 5 or ly > 5:
                if idx > 4:  # Skip head keypoints (0-4)
                    cv2.circle(frame, (lx, ly), 3, (255, 0, 0), -1)
# Zone Logic
        # Check if person is inside the zone
        person_in_zone = any_body_point_in_polygon(landmarks, state['polygon_points'])

        # Check if person is completely outside the zone
        person_completely_outside = is_person_completely_outside_zone(landmarks,
state['polygon_points'])

        box_center = ((x1 + x2) // 2, (y1 + y2) // 2)

        # Track people between frames
        person_id = None
        min_distance = float('inf')
        # Determine if this person matches a previous person based on proximity
        for pid, tracker in state['person_trackers'].items():
            last_x, last_y = tracker['last_seen']
            distance = np.hypot(last_x - box_center[0], last_y - box_center[1])
            if distance < 80 and distance < min_distance:
                person_id = pid
                min_distance = distance

        if person_id is None:
            person_id = max(state['person_trackers'].keys(), default=-1) + 1

        prev_tracker = state['person_trackers'].get(person_id, {
```

```
            'currently_in_zone': False,
            'completely_exited': True,
            'last_seen': box_center,
            'frames_outside': 0  # Count frames completely outside for stability
        })

        # Update tracker state
        currently_in_zone = person_in_zone
        completely_exited = prev_tracker['completely_exited']
        frames_outside = prev_tracker['frames_outside']

        # If person is completely outside, increment outside counter
        if person_completely_outside:
            frames_outside += 1
            # Mark as completely exited after being outside for 3 consecutive frames
            if frames_outside >= 3:
                completely_exited = True
        else:
            frames_outside = 0

        # Entry detection
        # Count entry: person enters zone AND they had previously completely exited
        if currently_in_zone and completely_exited and not prev_tracker['currently_in_zone']:
            state['cross_count'] += 1
            completely_exited = False

        new_person_trackers[person_id] = {
            'currently_in_zone': currently_in_zone,
            'completely_exited': completely_exited,
            'last_seen': box_center,
            'frames_outside': frames_outside
        }
        status_color = (0, 255, 0) if currently_in_zone else (255, 0, 0)


    state['person_trackers'] = new_person_trackers

    # Draw polygon points and boundary
    for pt in state['polygon_points']:
        cv2.circle(frame, pt, 6, (0, 0, 255), -1)
    if len(state['polygon_points']) == 4:
        cv2.polylines(frame, [np.array(state['polygon_points'], np.int32)], isClosed=True, color=(0,
0, 255),
                      thickness=2)
```

```
        cv2.putText(frame, "ZONE SET", (10, 100), cv2.FONT_HERSHEY_SIMPLEX, 0.6, (0, 0,
255), 2)
    else:
        cv2.putText(frame, f"Click {4 - len(state['polygon_points'])} more points", (10, 100),
cv2.FONT_HERSHEY_SIMPLEX,
                    0.6, (0, 255, 255), 2)

    # Draw buttons
    cv2.rectangle(frame, record_button_pos,
                  (record_button_pos[0] + button_size[0], record_button_pos[1] + button_size[1]),
                  (0, 0, 255) if recording else (200, 200, 200), -1)
    cv2.putText(frame, "REC..." if recording else "RECORD",
                (record_button_pos[0] + 10, record_button_pos[1] + 28),
                cv2.FONT_HERSHEY_SIMPLEX, 0.7, (255, 255, 255), 2)

    cv2.rectangle(frame, reset_button_pos,
                  (reset_button_pos[0] + button_size[0], reset_button_pos[1] + button_size[1]),
                  (100, 100, 100), -1)
    cv2.putText(frame, "RESET",
                (reset_button_pos[0] + 15, reset_button_pos[1] + 28),
                cv2.FONT_HERSHEY_SIMPLEX, 0.7, (255, 255, 255), 2)

    # Display count
    cv2.putText(frame, f"Entries: {state['cross_count']}", (10, 450),
                cv2.FONT_HERSHEY_SIMPLEX, 0.8, (0, 255, 0), 2)

# Save Video if recordi g
    if recording and video_writer:
        video_writer.write(frame)
# Display live video
    cv2.imshow('Camera View', frame)
# Press 'q' to quit
    if cv2.waitKey(1) & 0xFF == ord('q'):
        break

if video_writer:
    video_writer.release()
cap.release()
cv2.destroyAllWindows()
```
